# 25-Hydroxyvitamin D_3_ Levels, BsmI Polymorphism and Insulin Resistance in Brazilian Amazonian Children

**DOI:** 10.3390/ijms160612531

**Published:** 2015-06-03

**Authors:** Fernanda Cobayashi, Bárbara Hatzlhoffer Lourenço, Marly Augusto Cardoso

**Affiliations:** Department of Nutrition, School of Public Health, University of São Paulo, Avenida Dr. Arnaldo 715, CEP 01246-904 São Paulo, Brazil; E-Mails: fernanda.cobayashi@gmail.com (F.C.); barbaralourenco@usp.br (B.H.L.)

**Keywords:** vitamin D, genetic polymorphism, children, nutrition, insulin resistance

## Abstract

Vitamin D is associated with a wide range of other functions beyond bone development. We evaluated the factors associated with 25-hydroxyvitamin D levels in 974 children aged ≤10 years and the impact of BsmI polymorphism of the vitamin D receptor (VDR) gene (rs1544410) on metabolic parameters in a subsample (n: 430) with a follow-up 2 years later from the initial population-based cross-sectional study. Multiple linear regression models were used in the analyses. The prevalence (95% CI) of vitamin D deficiency, insufficiency and sufficiency of children was 11.1% (9.2–13.2), 21.8% (19.2–24.5) and 67.2% (64.1–70.1), respectively. Overall, 23% of the variation in serum 25-hydroxyvitamin D concentrations was accounted for by BsmI polymorphism β = −0.053 (95% CI) (−0.100, −0.006), maternal schooling (≥9 years) β = 0.100 (0.039, 0.161), serum vitamin E β = 0.478 (0.381, 0.574), total cholesterol concentration β = 0.232 (0.072, 0.393) and serum folate β = 0.064 (0.013, 0.115). BsmI polymorphism was positively associated with HOMA-IR β = 0.122 (0.002, 0.243) and fasting glucose concentration β = 1.696 (0.259, 3.133). In conclusion, variables related to socioeconomic level, the presence of the allele risk for BsmI and other nutrient concentrations were associated with serum 25-hydroxyvitamin D concentrations. Our results suggest that BsmI polymorphism is correlated with metabolic outcomes.

## 1. Introduction

Vitamin D is a steroid hormone known by its essential role in bone metabolism [1,2]. Receptors of its biologically active form 1,25(OH)_2_D can be found in most tissues and cells in the body. Previous studies found that the actions of vitamin D are mediated by the vitamin D receptor (VDR), a nuclear hormone receptor which modulates genetic transcription assisting in calcium absorption or bone formation proteins such calcium binding proteins and osteocalcin [3]. In addition, VDR is expressed in many tissues, and hence, other metabolic functions can be cited, such as the regulation of cell proliferation and differentiation and modulation of the immune response [4].

Despite the importance of the sun for vitamin D synthesis, the prevalence of deficiency or insufficiency in children and adolescents is high even in sunny regions [2,5]. There is a great variability in the prevalence of vitamin D deficiency among populations owing to the threshold defined, geographical location, lifestyle, fortified food intake and season [6]. Considering deficiency to be <50 nmol/L, 9% of children were vitamin D deficient in Denmark [7], 24% in Mexico [8], 41.4% in northern Algeria [9] and 45.9% in Italy [10]. Brazil lacks prevalence studies on vitamin D deficiency among preschool age children.

The optimum threshold for serum 25-hydroxyvitamin D concentration is still a matter of controversy in children, owing to sparse outcome data. Prolonged and severe vitamin D deficiency may impair bone metabolism, leading to the occurrence of rickets in children [6]. Previous cross-sectional studies have demonstrated that concentrations of calcium, alkaline phosphatase, and phosphorus may be very low in vitamin D deficient children (<50 nmol/L) [5], but other studies, focusing on the relationship between fractures in childhood and low serum 25-hydroxyvitamin D concentrations, are inconsistent [11]. Age, race, socioeconomic status, body mass index, season, sunlight exposure, eating habits, and season are also associated with low 25-hydroxyvitamin D levels [10,12,13].

Other factors, such as the joint effects of genetic polymorphism and micronutrients status on serum 25-hydroxyvitamin D concentrations, could play an important role, but investigations are scarce in pediatric populations.

The single nucleotide polymorphisms (SNPs) in the VDR gene have been associated with specific health outcomes, such as cancer, type 1 diabetes and factors related to metabolic syndrome [14,15,16]. They also may be involved in serum 25-hydroxyvitamin D concentrations. Santos *et al.* [17], studying Brazilian girls aged 7–18 years, observed that BsmI, ApaI and TaqI wild variants of the VDR gene were associated with low 25-hydroxyvitamin D levels. Conversely, Valtuena *et al.* [18] did not find any association between VDR genetic polymorphism (rs1544410) and serum 25-hydroxyvitamin D concentrations in adolescents.

The aim of this study was to evaluate the prevalence and associated factors of vitamin D deficiency in schoolchildren living in the Brazilian Amazon and to investigate the impact of VDR gene polymorphisms on metabolic parameters.

## 2. Results

### 2.1. Characteristics of Children

Of the 1225 participants initially enrolled, serum 25-hydroxyvitamin D concentrations were measured for 974 children (79.5% of those eligible). Of these, the mean age was 5.4 (SD 2.8) years (range: 2.8 months to 10.4 years). About 10% of children under 25 months of age were exclusively breastfed until six months; the median age of exclusive breastfeeding was 60 days of age. Table 1 shows the characteristics of these children. Almost 70% of mothers had less than 9 years of schooling. The prevalence of stunting (11.0%) or being overweight or obese (30.1%) was higher in children ≤24 months. Vitamin A deficiency was found in 15.1% of children. The prevalence of vitamin D deficiency, insufficiency and sufficiency of all children was 11.1% (95% CI: 9.2, 13.2), 21.8% (95% CI: 19.2, 24.5) and 67.2% (95% CI: 64.1, 70.1), respectively.

**Table 1:** Characteristics of children included in the study according to age group (Acrelândia, Brazil, 2007).

Variables	All (*n* 974) ^a^	<25 Months (*n* 158)	25 to <60 Months (*n* 287)	≥60 Months (*n* 529)
***Socio demographic characteristics*, *n* (%)**				
Child’s sex				
Male	497 (51.0)	86 (54.4)	146 (50.9)	265 (50.1)
Race/ethnicity				
White	93 (10.3)	15 (10.1)	31 (11.5)	47 (9.8)
Black	45 (5.0)	8 (5.4)	11 (4.1)	26 (5.4)
Brown	762 (84.7)	126 (84.6)	228 (84.4)	408 (84.2)
Maternal schooling (<9 years)	767 (69.2)	135 (65.8)	218 (65.3)	414 (72.6)
***Children’s characteristics*, *n* (%)**				
Low birth weight (<2500 g)	46 (5.3)	5 (3.4)	12 (4.5)	29 (6.4)
Stunting	49 (5.1)	17 (11.0)	12 (4.2)	20 (4.0)
Overweight or obesity	143 (14.8)	47 (30.1)	44 (15.4)	52 (9.9)
***Biochemical nutritional indicators*, *n* (%)**				
Serum 25-hydroxyvitamin D (nmol/L)				
Median (IQR) ^b^	66 (86–105)	60 (87.5–120)	61 (85–105)	69 (87–104)
<50	108 (11.0)	23 (14.6)	39 (13.6)	46 (8.7)
50–75	212 (21.8)	28 (17.7)	67 (23.3)	117 (22.1)
≥75	654 (67.2)	107 (67.7)	181 (63.1)	366 (69.2)
Serum vitamin A (µmol/L)				
Median (IQR) ^b^	0.7 (1.1–1.5)	0.8 (1.1–1.5)	0.9 (1.1–1.5)	0.9 (1.1–1.4)
<0.70	80 (15.1)	24 (15.2)	42 (14.6)	80 (15.1)
Serum vitamin E (µmol/L)				
Median (IQR) ^b^	14.4 (17.4–20.9)	14.7 (18.4–22.6)	14.3 (17.1–20.6)	14.4 (17.2–20.6)
<11.6	85 (8.7)	19 (12.0)	27 (9.4)	39 (7.4)
Serum folate (median, nmol/L)				
≥23.7	463 (47.7)	73 (46.2)	136 (47.5)	254 (48.2)
Total cholesterol (mg/dL)				
Median (IQR) ^b^	132 (151–172)	132 (151–180)	135 (155–175)	132 (149–168)
***Morbidities*, *n* (%)**				
C-reactive protein >5 mg/L	90 (9.4)	21 (13.5)	26 (9.4)	43 (8.2)
Diarrhea in the past 15 days	214 (22.2)	67 (42.7)	70 (24.6)	77 (14.7)

^a^ Total may be less because of missing values; ^b^ IQR, Interquartile ranges.

### 2.2. Gene Allele Distributions

Increased risk allele for low serum 25-hydroxyvitamin D concentrations according to the present study was observed only for the VDR BsmI gene polymorphism. Mean differences in 25-hydroxyvitamin D levels for FokI, ApaI, TaqαI and Cdx2 were not observed according to allele. The distribution of VDR polymorphisms is presented in Table 2.

**Table 2:** Vitamin D receptor polymorphisms distribution in 974 children aged <10 years (Acrelândia, Brazil, 2007).

Gene	SNP	Wild (A)	Mutant (a)	Frequency (%) AA/Aa/aa ^a^
FokI	rs2228570	G	A	50.3/39.5/10.2
BsmI	rs1544410	C	T ^b^	27.9/63.0/9.1
ApaI	rs7975232	A	C	24.0/65.8/10.2
TaqαI	rs731236	A	G	29.9/61.0/9.1
Cdx2	rs11568820	G	A	27.5/65.3/7.2

^a^ Total may be less because of missing values; ^b^ Increased risk allele for low serum 25-hydroxyvitamin D concentration according to the present study.

### 2.3. Factors Associated with Serum 25-Hydroxyvitamin D Concentrations at Baseline

Table 3 shows the factors associated with 25-hydroxyvitamin D adjusted for sex and race/ethnicity for all children. The final model explained approximately 23% of the variability in serum 25-hydroxyvitamin D concentrations. VDR polymorphism BsmI (allele in addictive model) was negatively associated with 25-hydroxyvitamin D, whereas maternal schooling (≥9 years), serum vitamin E, total cholesterol concentration and serum folate above median (≥23.6 nmol/L) were positively associated with serum 25-hydroxyvitamin D concentrations. For the linear regression analysis, the serum vitamin E/total cholesterol ratio was not used owing to the better adjustment of final R squared in the final model.

**Table 3:** Factors associated with serum 25-hydroxyvitamin D concentrations in urban Amazonian children <10 years old (Acrelândia, Brazil, 2007).

	Serum 25-Hydroxyvitamin D Concentrations (N 974) ^a^						
Independent Variables	Crude β-Coefficient	95% CI	*p* ^b^	Adjusted β-Coefficient	95% CI	*p* ^b^	*R*^2 c^
**Polymorphisms**							0.228
BsmI (rs1544410)	−0.070	−0.132, −0.008	0.026	−0.053	−0.100, −0.006	0.025	
**Socioeconomic status**							
Maternal schooling (years)							
<9	*Reference*			*Reference*			
≥9	0.075	0.020, 0.130	0.007	0.100	0.039, 0.161	0.001	
**Biochemical indicators**							
Serum vitamin E (µmol/L)	0.538	0.466, 0.611	<0.001	0.478	0.381, 0.574	<0.001	
Total cholesterol (mg/dL)	0.640	0.506, 0.774	<0.001	0.232	0.072, 0.393	0.005	
Serum folate (median, nmol/L)							
<23.6	*Reference*			*Reference*			
≥23.6	0.062	0.012, 0.112	0.015	0.064	0.013, 0.115	0.013	

^a^ Dependent and independent variables were log-transformed before analysis; ^b^ All models were adjusted by sex, age and race/ethnicity; ^c^ Final adjusted R-squared. Interaction term: BsmI x vitamin E (*p* = 0.453); BsmI x total cholesterol (*p* = 0.740).

In additional analyses, we also investigated the factors associated with vitamin D deficiency (<50 nmol/L) (Supplementary Material, Table S1). The factors associated with vitamin D deficiency that remained significant in the model were wealth concentration, low birth weight and vitamin E insufficiency. VDR polymorphism BsmI gene lost its significance in the stratified analysis. No association was found in the insufficient vitamin D group of children.

### 2.4. Impact of VDR BsmI Polymorphism on Metabolic Parameters

A total of 311 children revisited in 2009 with complete information on insulin and glucose concentrations and blood pressure measurements were evaluated to investigate the associations of VDR BsmI gene polymorphism with metabolic parameters (Table 4). Glucose concentrations and HOMA-IR were positively associated with the VDR BsmI SNP (coded additively for the minor allele) after full adjustment for age, sex, race/ethnicity, BMI-for-age Z-scores, Tanner stage and baseline household wealth. The interactions between the VDR BsmI SNP and 25-hydroxyvitamin D on the metabolic parameters were not significant (*p* > 0.05).

**Table 4:** Association of Vitamin D receptor (VDR) BsmI gene polymorphism with metabolic parameters (Acrelândia, Brazil, 2007–2009).

	VDR BsmI Gene Polymorphism						
Variables	Adjusted β-Coefficient ^a^	95% CI	*R*^2^	Fully Adjusted β-Coefficient ^b^	95% CI	*R*^2^	*p* for Interaction between BsmI x 25-Hydroxyvitamin D
Insulin concentration	0.095	−0.028; 0.218	0.168	0.104	−0.008; 0.216	0.319	0.323
HOMA-IR	0.110	−0.020; 0.242	0.170	0.122	0.002; 0.243	0.316	0.336
Glucose (mg/dL)	1.502	0.064; 2.940	0.101	1.696	0.259; 3.133	0.113	0.591
Systolic blood pressure (mmHg)	−0.667	−2.421; 1.085	0.020	−0.646	−2.413; 1.119	0.021	0.625
Diastolic blood pressure (mmHg)	−0.180	−1.402; 1.042	0.012	−0.125	−1.362; 1.111	0.003	0.678
25-hydroxyvitamin D (nmol/L)	0.007	−0.059; 0.073	0.012	0.100	−0.056; 0.076	0.001	--

VDR BsmI polymorphism was coded additively for the minor allele. HOMA-IR: homeostasis model assessment index of insulin resistance. *p* values were calculated using linear regression models. Insulin levels, HOMA-IR and 25-hydroxyvitamin D were log-transformed before analysis. ^a^ Model was adjusted for age, sex and race/ethnicity; ^b^ Model was adjusted for age, sex, race/ethnicity, BMI-for-age Z-scores, Tanner stage and baseline household wealth; models for systolic and diastolic blood pressure were additionally adjusted for the child’s height.

## 3. Discussion

This study found that the overall prevalence of vitamin D deficiency was 11%, 14.6%, 13.6% and 8.7% for children aged <25, 25 to <60, and ≥60 months, respectively, while vitamin D insufficiency or deficiency (<75 nmol/L) reached almost 33% for all the children. Considering the same cutoff used in our analyses, the prevalence of vitamin D deficiency among other studies ranged from 8% to 29.9% in children [7,8,9]. Factors such as threshold, season, age group, vitamin supplementation and geographical location among others are responsible for the great variation in the prevalence [6]. The median serum 25-hydroxyvitamin D concentrations in the whole group of the present study was 66 nmol/L, lower than that found in Mexican children (94.6 nmol/L) [8] and in Algerian children (71.4 nmol/L) [9].

Despite its location in a sunny region at low latitude (9°49ʹS), our study children were at increased risk of vitamin D inadequacy. This region receives a consistent daily solar radiation throughout the year. Winter or the rainining season is considered to be from November to April when heavy rainfalls occur, but even during this period, days entirely without sunshine are rare. A seasonal variation in serum 25-hydroxyvitamin D concentrations has been decribed in São Paulo [19], but this has not been observed at the same latitude in Brazil in previous published papers [20].

Although controversy exists about which 25-hydroxyvitamin D levels would be considered adequate [21], previous studies conducted in children have demonstrated that concentrations of calcium, alkaline phosphatase, and phosphorus were very low in vitamin D deficient children (<50 nmol/L) [5], whereas serum 25-hydroxyvitamin D concentrations above 75 nmol/L are positively associated with higher bone mineral content and areal bone mineral density [22].

In our study, the factors independently associated with serum 25-hydroxyvitamin D concentrations were the presence of VDR BsmI gene polymorphism, which was negatively associated with 25-hydroxyvitamin D, whereas higher maternal schooling, vitamin E, total cholesterol and higher levels of serum folate were positively associated.

Of all genes studied, we observed that only the VDR BsmI gene polymorphism was associated with 25-hydroxyvitamin D. Although genetics determines serum 25-hydroxyvitamin D concentrations in males as demonstrated by Arguelles *et al.* [23] in their study with Chinese adolescent twins, the few studies that have been conducted in children to investigate the influence of VDR gene polymorphisms on vitamin D levels have reported divergent results. Santos *et al.* [17], studying a group of Brazilian girls aged 7–18 years, found that VDR wild-type genotypes BsmI, ApaI and TaqI were significantly associated with lower 25-hydroxyvitamin D levels, but others did not find any associations [18]. More studies will be required to elucidate the contribution of VDR genetic polymorphisms on serum 25-hydroxyvitamin D concentrations in apparently healthy children.

Vitamin A deficiency in the current study was found in 15% of the participants, which is considered a moderate public health problem by the WHO [24], whereas vitamin E insufficiency was almost 9%. We also observed that serum total cholesterol and folate above the median concentrations were positively associated with 25-hydroxyvitamin D. It is noteworthy that our total study sample consisted of low-income children who are frequently exposed to infections, and have insufficient basic sanitation or water treatment and poor diet resulting in a compromised micronutrient status. Our results are in agreement with other studies regarding the association between low socioeconomic level and vitamin D deficiency [9,25].

As previously described, Amazonian children have a poor quality diet. At an early age, these children as reported in our study population had a low intake of fruit, vegetables, and animal-derived food, with substantial consumption of unhealthy foods such as processed foods high in sugars and fats [26,27]. It is important to note that our study children had no access to foods fortified with vitamin D and did not take any vitamin or mineral supplements [26]. We evaluated the relation between animal derived-foods intake and 25-hydroxyvitamin D levels and no significant associations were found. This lack of association is most likely due to the homogenous low-level consumption of the study population. The frequency of food intake such as meat, milk, eggs and fish, which are known to contain vitamin D metabolites [28], was low. Only 50% of the children reported milk or meat consumption ≥2 per day, 34% of them consumed eggs ≥1 per day and only 15% of the sample consumed fish ≥1 per week. The lack of association between intake of vitamin D from food and serum 25-hydroxyvitamin D concentrations has also been reported in other studies [28,29].

Interestingly, when the prevalence ratio analyses were performed considering only vitamin D deficient children (<50 nmol/L), the most important factors associated with vitamin D deficiency that remained significant in the final model were wealth index, low birth weight, and vitamin E insufficiency (supplementary material). VDR BsmI gene polymorphism was no longer significant. These results clearly show that in our study children, socioeconomic conditions as well as nutritional status are more likely to influence serum 25-hydroxyvitamin D concentrations than genetic factors.

The association between 25-hydroxyvitamin D and VDR BsmI gene polymorphism lead us to investigate whether the VDR BsmI gene polymorphism was associated with metabolic parameters. According to our results performed with a subsample of the same children reexamined 2 years later, VDR BsmI gene polymorphism influenced glucose concentrations and HOMA-IR even after adjustments for BMI-for-age Z-score and puberty, among other covariates. Nevertheless, any interaction between BsmI and 25-hydroxyvitamin D was significant for each metabolic outcome studied (insulin and glucose concentrations, HOMA-IR, systolic and diastolic blood pressure).

Oh and Barrett-Connor [30], in their study with nondiabetic Caucasians, observed that bearers of the bb genotype of BsmI polymorphism had significantly higher levels of HOMA-IR compared with those with BB and Bb genotypes. However, studies considering subjects with or without metabolic syndrome did not find associations between BsmI polymorphism and insulin resistance and fasting glycemia [31].

Insulin resistance is an important component of the metabolic syndrome, and children with insulin resistance are at increased risk for type 2 diabetes [32]. VDR is expressed in the pancreatic beta cell and the presence of VDR polymorphism may influence insulin secretion, as previously demonstrated [33,34,35]. Consequently, the presence of those polymorphisms could lead people to an increased risk for type 2 diabetes [36]. The role of VDR gene polymorphism and type 2 diabetes has been studied, but the results are not consistent among different populations. Regarding VDR BsmI gene polymorphism, two recent meta-analysis studies did not find an association with type 2 diabetes risk in Asian or Caucasian populations [36,37]. Heterogeneity among populations and the lack of studies to elucidate the genetic differences might explain the discrepancies between the studies [16].

Some limitations of the present study should be considered. Because of its cross-sectional design, caution should be taken in interpreting the findings. In addition, we did not investigate mutations in genes related to the transport of vitamin D, the time of solar exposure or the indicators of bone mass owing to related logistic and methodological issues. Despite these limitations, this study has yielded estimates of factors associated with serum 25-hydroxyvitamin D concentrations, including the joint effect of VDR genetic polymorphisms, in a population-based study with children. In addition, to our knowledge, this is the first Brazilian study that evaluated the 25-hydroxyvitamin D levels in a group of representative children living in a poor urban area.

## 4. Experimental Section

### 4.1. Study Population

This population-based study was conducted in Acrelândia (9°49ʹS, 66°53ʹW), a frontier town located 112 km from Rio Branco, the capital of the State of Acre in the Western Brazilian Amazon region. In 2007, the town had 11,520 inhabitants at baseline, of which 44% resided in an urban area, comprising mainly migrants from southeastern and southern regions of Brazil engaged in commercial agriculture and cattle farming.

Sampling strategies and field procedures have been previously reported [38]. Briefly, in December 2007, a baseline cross-sectional survey on child health and nutrition enrolled 1225 children up to 10 years of age (98.0% of those eligible for the study). In the present analyses, in order to evaluate baseline factors associated with the nutritional status of vitamin D, a structured questionnaire was administered through face-to-face interview with the mothers or guardians of children to collect data on demographic characteristics, socioeconomic status, environmental factors, reproductive health variables, infant feeding practices, amount spent with outdoor activities, and recent morbidities. After the interview, children were invited to visit the local family health clinic, where research assistants carried out a physical examination and trained phlebotomists obtained a venous blood sample from all children.

A follow-up assessment was carried out in December 2009 and 909 children of those included at baseline (74.2%) were identified. To explore the longitudinal impact of VDR polymorphisms on metabolic parameters, we focused on children aged 4–10 years with complete VDR SNP information in 2007 (*n* = 430; 73.74% of those initially enrolled in this age group). In 2009, these children were invited for a clinical examination for pubertal development evaluation, anthropometric and blood pressure measurements, and blood sample collection.

The study was performed according to the guidelines laid down in the Declaration of Helsinki and all parents or guardians of children provided written informed consent prior to enrolment. This study protocol was approved by the institutional review board of the School of Public Health, University of São Paulo, Brazil (No. 1681/07).

### 4.2. Anthropometric Assessment

Trained research assistants following standardized procedures using calibrated equipment [39] performed anthropometric measurements. At baseline, among children aged <24 months, recumbent length was measured using a locally made infant measuring board, and weight was measured with an electronic pediatric scale (model 1583; Tanita, Tokyo, Japan). Among children aged ≥24 months, at baseline and follow-up assessments, height was measured using a stadiometer (model 208; SECA, Hamburg, Germany), and weight was measured using an electronic scale (model HS-302; Tanita, Tokyo, Japan). Each measurement was repeated and the mean value was calculated. BMI was computed as weight (kg) divided by height (m^2^). Z-scores for length/height-for-age (HAZ) and BMI-for-age (BAZ) were calculated according to WHO guidelines [40]. Stunting was defined as HAZ < −2, and overweight as BAZ > 1 [40,41].

### 4.3. Dietary Assessment

For children <24 months, a diet history [27] was collected by trained nutritionists. The interviewers were provided with household measures to help mothers or guardians estimate the habitual amounts of food or beverages. For children ≥24 months, a food frequency questionnaire, based on a validation study in this area [42], was used to estimate the frequency of food consumption (fruit, green vegetables, root vegetables, dairy, beans, meat, eggs and fish) within the last month. The World Food Dietary Assessment System (version 2.0; University of California, Berkeley, CA, USA) was used to estimate food intake.

### 4.4. Clinical Examinations and Biochemical Measures

At baseline in 2007, a sample (approximately 5 mL) of fasting venous blood was collected from children during the visit to the local family health clinic of Acrelândia. Serum and plasma samples were shipped to São Paulo on dry ice and frozen at −70 °C until further analysis. Because limited sample volumes were available, not all laboratory analyses were performed for all children.

Serum 25-hydroxyvitamin D_3_, retinol and tocopherol concentrations were determined by standard high-performance liquid chromatography methods (Shimadzu, Kyoto, Japan). The cutoffs used for 25-hydroxyvitamin D_3_ were the following: <50, 50–75 and ≥75 nmol/L [1]; concentrations <0.70 µmol/L defined vitamin A deficiency [23]; and vitamin E insufficiency was defined as <11.6 µmol/L [43]. Plasma C-reactive protein (CRP) concentration was measured using the Immulite high-sensitivity chemiluminescent assay (DPC, Los Angeles, CA, USA). The cutoff for high CRP was >5 mg/L as an indicator of inflammation [44]. Serum lipid fractions were measured enzymatically using an automatic device (ADVIA 1650; Bayer, East Walpole, MA, USA).

During the follow-up assessment in 2009, trained research assistants ascertained pubertal development according to the Tanner stages [45] and measured blood pressure after 5 min of rest using an automatic digital device with appropriate cuff sizes. We used the mean of three measurements of systolic and diastolic blood pressure. A sample (approximately 5 mL) of fasting venous blood was collected from the children and handled in the same way as described at baseline. Glucose concentrations were determined with the automatic enzymatic method using hexokinase. Insulin concentrations were determined by fluoroimmunoassay. The homeostasis model assessment index of insulin resistance (HOMA-IR) was calculated as insulin (mUl/L) × glucose (mmol/L)/22.5 [46].

### 4.5. Genotyping

DNA was extracted from EDTA-containing whole blood aliquots using DNA kits (Qiagen, Hilden, Germany). SNP genotyping was performed using allele-specific polymerase chain reaction (PCR) with the molecular beacons [47], under contract by Prevention Genetics (Marshfield, WI, USA). SNPs included those in the VDR gene: BsmI (rs1544410), TaqI (rs731236), ApaI (rs7975332), FokI (rs2228570) and Cdx2 (rs11568820). The homogeneous assay used two tailed allele-specific primers, a common reverse primer, and two different fluorescently labeled universal primers in a single-well reaction. Submicroliter PCR reactions were carried out with Array Tape instrumentation and allele calls were generated based on clustering of fluorescent signals [48]. The internal quality of genotype data was assessed by typing 10% of blinded samples in duplicate; the resulting concordance was >99%. Allelic and genotypic frequencies for each SNP were calculated from the Hardy-Weinberg equilibrium (*p* > 0.05) using an available online tool [49].

### 4.6. Statistical Analysis

To investigate baseline factors associated with the nutritional status of vitamin D in 2007, vitamin D was considered as the outcome of interest in cross-sectional analysis as a continuous variable (natural log-transformed) and according to the classification for deficiency (<50 nmol/L). Explanatory variables comprised socioeconomic status, children’s characteristics, biochemical indicators, morbidities and VDR polymorphisms, assuming that each SNP was independently associated with risk according to an additive genetic model. Principal component analysis was used to derive a wealth index representing a proxy of household income [50], based on the presence of 12 household assets as described elsewhere [38]. The household wealth index was examined in quartiles. Maternal schooling was categorized as <9 years *vs.* ≥9 years. Child’s birth weight was categorized as <2500 g *vs.* ≥2500 g. Morbidities taken into account were diarrhea in the past 15 days and CRP concentration (≤5 *vs.* >5 mg/L).

For the descriptive analyses at baseline, children were stratified into age categories (≤25, 25 to <60 and ≥60 months). Linear regression analyses were conducted for baseline serum 25-hydroxyvitamin D concentrations and each of the potential explanatory variables. Serum vitamin A, vitamin E and total cholesterol concentrations were natural log-transformed before linear regression analysis. Prevalence ratios (PR) and 95% confidence intervals (95% CI) were also obtained for the factors associated with baseline vitamin D deficiency (<50 nmol/L) using multiple Poisson regression models with robust variance. For both analytical approaches, covariates were selected for multiple regression models following a hierarchical conceptual approach [51] and considering crude associations with the outcome at *p* < 0.20. Then, at each level of determination, covariates were retained in the models if associated with the outcome at *p* < 0.10, or if categories followed a dose-response pattern, or if inclusion in the model changed R2 or PR by 10% or more. Missing observations were included in the multiple models by creating missing-value categories. The outcome of the interaction terms between VDR and biochemical measures were tested in the models.

Of all VDR polymorphisms analyzed, VDR genotype BsmI (rs1544410) was associated with 25-hydroxyvitamin D in our study population. Thus, in longitudinal analysis to verify the impact of VDR polymorphisms on metabolic parameters, we fitted linear regression models with VDR gene BsmI polymorphism (assessed in 2007) as the main exposure and glucose and insulin concentrations, insulin resistance, and systolic and diastolic blood pressure (measured in school-aged children in 2009) as the outcomes of interest. Initially, estimates were adjusted in crude models for age, sex and race/ethnicity. Models for systolic and diastolic blood pressure were also adjusted for the child’s height at the follow-up assessment. Finally, fully adjusted models for each metabolic parameter considered all covariates in the crude models with further control for baseline household wealth, BMI-for-age Z scores and Tanner stage. Insulin, HOMA-IR, and serum 25-hydroxyvitamin D concentrations were log-transformed before analysis. Interactions between VDR BsmI polymorphism and 25-hydroxyvitamin D were also assessed. *p* values reported are two-sided. All analyses were performed using Stata Corp software, College Station, TX, USA, version 13.0.

## 5. Conclusions

In conclusion, our findings showed that urban Amazonian children are at risk of vitamin D deficiency. VDR BsmI gene polymorphism was negatively associated with serum 25-hydroxyvitamin D concentrations. However, higher maternal schooling, serum vitamin E, total cholesterol and serum folate concentrations contributed positively with 25-hydroxyvitamin D. In addition, the VDR BsmI was significantly associated with insulin resistance and fasting glucose concentrations. Considering the wide range of other functions in which vitamin D can be involved, effective public health programs are necessary to prevent micronutrient deficiencies during childhood. Finally, more long-term studies are necessary to elucidate the joint effect of this polymorphism with metabolic parameters, environmental factors and the critical metabolic implications.

## Supplementary Materials

Supplementary materials can be found at http://www.mdpi.com/1422-0067/16/06/12531/s1.

## Appendix

The ACTION (ACre nutriTION) Study Team: Pascoal Torres Muniz, Orivaldo Florencio Souza, Cristieli Sergio de Menezes Oliveira, Thiago Santos de Araujo (Department of Health Sciences, Federal University of Acre, Rio Branco, Brazil); Suely de Godoy Agostinho Gimeno, Luciana Yuki Tomita (Department of Preventive Medicine, Federal University of São Paulo, São Paulo, Brazil); Marcelo Urbano Ferreira (Institute of Biomedical Sciences, University of São Paulo, São Paulo, Brazil); Kézia K. G. Scopel (Department of Parasitology, Immunology and Microbiology, Federal University of Juiz de Fora, Juiz de Fora, Brazil); Barbara Hatzlhoffer Lourenç̧o, Pablo Secato Fontoura, Fernanda Serra Granado, Fernanda Cobayashi, Rosangela Aparecida Augusto, Marly Augusto Cardoso (Department of Nutrition, School of Public Health, University of São Paulo, São Paulo, Brazil).

## References

[1] Holick M.F. (2007). Vitamin D deficiency. N. Engl. J. Med..

[2] Koo W., Walyat N. (2013). Vitamin D and skeletal growth and development. Curr. Osteoporos. Rep..

[3] Ferrari S., Bonjour J.P., Rizzoli R. (1998). The vitamin D receptor gene and calcium metabolism. Trends Endocrinol. Metab..

[4] Uitterlinden A.G., Fang Y., van Meurs J.B.J., Pols H.A.P., van Leeuwen J.P.T.M. (2004). Genetics and biology of vitamin D receptor polymorphisms. Gene.

[5] Bener A., Al-Ali M., Hoffmann G.F. (2009). Vitamin D deficiency in healthy children in a sunny country: Associated factors. Int. J. Food. Sci. Nutr..

[6] Holick M.F., Binkley N.C., Bischoff-Ferrari H.A., Gordon C.M., Hanley D.A., Heaney R.P., Murad M.H., Weaver C.M. (2011). Evaluation, treatment, and prevention of vitamin D deficiency: An endocrine society clinical practice guideline. J. Clin. Endocrinol. Metab..

[7] Madsen K.H., Rasmussen L.B., Mejborn H., Andersen E.W., Mølgaard C., Nissen J., Tetens I., Andersen R. (2014). Vitamin D status and its determinants in children and adults among families in late summer in Denmark. Br. J. Nutr..

[8] Flores M., Macias N., Lozada A., Sánchez L.M., Díaz E., Barquera S. (2013). Serum 25-hydroxyvitamin D levels among Mexican children ages 2 y to 12 y: A national survey. Nutrition.

[9] Djennane M., Lebbah S., Roux C., Djoudi H., Cavalier E., Souberbielle J.C. (2014). Vitamin D status of schoolchildren in Northern Algeria, seasonal variations and determinants of vitamin D deficiency. Osteoporos. Int..

[10] Vierucci F., del Pistoia M., Fanos M., Gori M., Carlone G., Erba P., Massimetti G., Federico G., Saggese G. (2013). Vitamin D status and predictors of hypovitaminosis D in Italian children and adolescents: A cross-sectional study. Eur. J. Pediatr..

[11] Moon R.J., Harvey N.C., Davies J.H., Cooper C. (2014). Vitamin D and skeletal health in infancy and childhood. Osteoporos. Int..

[12] Black L.J., Burrows S.A., Jacoby P., Oddy W.H., Beilin L.J., Chan She Ping-Delfos W., Marshall C.E., Holt P.G., Hart P.H., Mori T.A. (2014). Vitamin D status and predictors of serum 25-hydroxyvitamin D concentrations in Western Australian adolescents. Br. J. Nutr..

[13] Angurana S.K., Angurana R.S., Mahajan G., Kumar N., Mahajan V. (2014). Prevalence of vitamin D deficiency in apparently healthy children in north India. J. Pediatr. Endocrinol. Metab..

[14] Gnagnarella P., Pasquali E., Serrano D., Raimondi S., Disalvatore D., Gandini S. (2014). Vitamin D receptor polymorphism FokI and câncer risk: A comprehensive meta-analysis. Carcinogenesis.

[15] Tizaoui K., Kaabachi W., Hamzaoui A., Hamzaoui K. (2014). Contribution of VDR polymorphisms to type 1 diabetes susceptibility: Systematic review of case-control studies and meta-analysis. J. Steroid Biochem. Mol. Biol..

[16] Al-Daghri N.M., Al-Attas O.S., Alkharfy K.M., Khan N., Mohammed A.K., Vinodson B., Ansari M.G., Alenad A., Alokail M.S. (2014). Association of VDR-gene variants with factors related to the metabolic syndrome, type 2 diabetes and vitamin D deficiency. Gene.

[17] Santos B.R., Mascarenhas L.P.G., Satler F., Boguszewski M.C.S., Spritzer P.M. (2012). Vitamin D deficiency in girls from South Brazil: A cross-sectional study on prevalence and association with vitamin D receptor gene variants. BMC Pediatr..

[18] Valtueña J., González-Gross M., Huybrechts I., Breidenassel C., Ferrari M., Mouratidou T., Gottrand F., Dallongeville J., Azzini E., Sioen I. (2013). Factors associated with vitamin D deficiency in European adolescents: The Helena study. J. Nutr. Sci. Vitaminol..

[19] Maeda S.S., Saraiva G.L., Hayashi L.F., Cendoroglo M.S., Ramos L.R., Correa M.P., Mesquita C.H., Lazaretti-Castro M. (2013). Seasonal variation in the serum 25-hydroxyvitamin D levels of young and elderly active and inactive adults in São Paulo, Brazil. Dermatoendocrinology.

[20] Linhares E.R., Jones D.A., Round J.M., Edwards R.H.T. (1984). Effect of nutrition on vitamin D status: Studies on healthy and poorly nourished Brazilian children. Am. J. Clin. Nutr..

[21] Heaney R.P., Holick M.F. (2011). Why the IOM recommendations for vitamin D are deficient?. J. Bone Miner. Res..

[22] Hazell T.J., Pham T.T., Jean-Philippe S., Finch S.L., El Hayek J., Vanstone C.A., Rodd C.J., Weiler H.A. (2014). Vitamin D status is associated with bone mineral density and bone mineral content in preschool-aged children. J. Clin. Densitom..

[23] Arguelles L.M., Langman C.B., Ariza A.J., Ali F.N., Dilley K., Price H., Liu X., Zhang S., Hong X., Wang B. (2009). Heritability and environmental factors affecting vitamin D status in rural Chinese adolescent twins. J. Clin. Endocrinol. Metab..

[24] World Health Organization (2009). Global Prevalence of Vitamin A Deficiency in Populations at Risk 1995–2005: WHO Global Database on Vitamin A Deficiency.

[25] Puri S., Marwaha R.K., Agarwal N., Tandon N., Agarwal R., Grewal K., Reddy D.H., Singh S. (2008). Vitamin D status of apparently healthy schoolgirls from two different socioeconomic strata in Delhi: Relation to nutrition and lifestyle. Br. J. Nutr..

[26] Augusto R.A., Cobayashi F., Cardoso M.A. (2014). Associations between low consumption of fruits and vegetables and nutritional deficiencies in Brazilian schoolchildren. Public Health Nutr..

[27] Garcia M.T., Granado F.S., Cardoso M.A. (2011). Complementary feeding and nutritional status of 6–24 month-old children in Acrelândia, Acre State, Western Brazilian Amazon. Cad. Saude Publica.

[28] Taylor C.L., Patterson K.Y., Roseland J.M., Wise S.A., Merkel J.M., Pehrsson P.R., Yetley E.A. (2014). Including food 25-Hydroxyvitamin D in intake estimates may reduce the discrepancy between dietary and serum measures of vitamin D status. J. Nutr..

[29] Absoud M., Cummins C., Lim M.J., Wassmer E., Shaw N. (2011). Prevalence and predictors of vitamin D insufficiency in children: A Great Britain population based study. PLoS ONE.

[30] Oh J.Y., Barrett-Connor E. (2002). Association between vitamin D receptor polymporphism and type 2 diabetes or metabolic syndrome in community-dwelling older adults: The Rancho Bernardo Study. Metabolism.

[31] Shuch N.J., Garcia V.C., Vívolo S.R.G.F., Martini L.A. (2013). Relationships between vitamin D receptor gene polymorphisms and the components of metabolic syndrome. Nutr. J..

[32] Global IDF/ISPAD Guideline for Diabetesin Childhood and Adolescence. http://www.idf.org/sites/default/files/Diabetes-in-Childhood-and-Adolescence-Guidelines.pdf.

[33] Hitman G.A., Mannan N., McDermott M.F., Aganna E., Ogunkolade B.W., Hales C.N., Boucher B.J. (1998). Vitamin D receptor gene polymorphisms influence insulin secretion in Bangladeshi. Asians Diabetes.

[34] Chiu K.C., Chuang L.M., Yoon C. (2001). The vitamin D receptor polymorphism in the translation initiation codon is a risk factor for insulin resistance in glucose tolerant Caucasians. BMC Med. Genet..

[35] Ortlepp J.R., Lauscher J., Hoffmann R., Hanrath P., Joost H.G. (2001). The vitamin D receptor gene variant is associated with the prevalence of type 2 diabetes mellitus and coronary artery disease. Diabet. Med..

[36] Li L., Wu B., Liu J., Yang L.B. (2013). Vitamin D receptor gene polymorphisms and type 2 diabetes: A meta-analysis. Arch. Med. Res..

[37] Zhu B., Zhao H., Ou C., Huang L., Li P., Lao M. (2014). Association of vitamin D receptor BsmI gene polymorphism with the risk of type 2 diabetes mellitus. J. Recept. Signal. Transduct..

[38] Cardoso M.A., Scopel K.K., Muniz P.T., Villamor E., Ferreira M.U. (2012). Underlying factors associated with anemia in Amazonian children: A population-based, cross-sectional study. PLoS ONE.

[39] Lohman T.G., Roche A.F., Martorell R. (1988). Anthropometric Standardization Reference Manual.

[40] World Health Organization (2006). Multicentre Growth Reference Study Group WHO child growth standards based on length/height, weight and age. Acta Paediatr..

[41] De Onis M., Lobstein T. (2010). Defining obesity risk status in the general childhood population: Which cut-offs should we use?. Int. J. Pediatr. Obes..

[42] Scagliusi F.B., Garcia M.T., Indiani A.L., Cardoso M.A. (2011). Relative validity of a food-frequency questionnaire developed to assess food intake of schoolchildren living in the Brazilian Western Amazon. Cad. Saúde Publica.

[43] Looker A.C., Underwood B.A., Wiley J., Fulwood R., Sempos C.T. (1989). Serum α-tocopherol levels of Mexican American, Cubans and Puerto Ricans aged 4–74 y. Am. J. Clin. Nutr..

[44] Thurnham D.I., McCabe L.D., Haldar S., Wieringa F.T., Northrop-Clewes C.A., McCabe G.P. (2010). Adjusting plasma ferritin concentrations to remove the effects of subclinical inflammation in the assessment of iron deficiency: A meta-analysis. Am. J. Clin. Nutr..

[45] Tanner J.M. (1962). Growth of Adolescents.

[46] Matthews D.R., Hosker J.P., Rudenski A.S., Naylor B.A., Treacher D.F., Turner R.C. (1985). Homeostasis model assessment: Insulin resistance and beta-cell function from fasting plasma glucose and insulin concentrations in man. Diabetologia.

[47] Myakishev M.V., Khripin Y., Hu S., Hamer D.H. (2011). High-throughput SNP genotyping by allele-specific PCR with universal energy-transfer-labeled primers. Genome Res..

[48] Rusch T.L., Dickinson W., Che J., Fieweger K., Chudyk J., Doktycz M.J., Yu A., Weber J.L. Instrumentation for Continuous Array Genotyping of Short Insert/Deletion Polymorphisms. Proceedings of the SPIE—Microarrays and Combinatorial Technologies for Biomedical Applications: Design, Fabrication, and Analysis.

[49] Rodrigues S., Gaunt T.R., Day I.N. (2009). Hardy-Weinberg equilibrium testing of biological ascertainment for Mendelian randomization studies. Am. J. Epidemiol..

[50] Filmer D., Pritchett L.H. (2001). Estimating wealth effects without expenditure data-or tear: An application to educational enrolments in states of India. Demography.

[51] Victora C.G., Huttly S.R., Fuchs S.C., Olinto M.T. (1997). The role of conceptual frameworks in epidemiological analysis: A hierarchical approach. Int. J. Epidemiol..

