# Supplementary material for: 25-Hydroxyvitamin D3 Levels, BsmI Polymorphism and Insulin Resistance in Brazilian Amazonian Children
Source: Int J Mol Sci. 2015 Jun 3;16(6):12531–46. doi: 10.3390/ijms160612531 (PMC4490459; doi:10.3390/ijms160612531)
Supplement: Supplementary file 1 [file ijms-16-12531-s001.pdf]

Supplementary Information

Table S1. Factors associated with vitamin D deficiency (<50 nmol/L) in urban Amazonian children under 10 years old, Acrelândia, Brazil, 2007.

	Crude			Adjusted *		
**Variables**	**PR**	**95%, (CI)**	***P***	**PR**	**95%, (CI)**	***P***
Socioeconomic status						
Wealth concentration (quartiles)						
1st	*Reference*			*Reference*		
2nd	1.82	1.06, 3.15	0.030	1.85	1.07, 3.20	0.027
3rd	1.65	0.95, 2.86	0.070	1.71	0.98, 2.95	0.054
4th	1.00	0.53, 1.87	0.989	1.14	0.60, 2.19	0.679
Maternal schooling (years)						
<9	*Reference*			*Reference*		
≥9	0.67	0.43, 1.05	0.087	0.71	0.44, 1.14	0.167
Child's characteristics						
Low birth weight (<2500 g)	1.88	1.01, 3.51	0.046	1.92	1.05, 3.51	0.033
Biochemical indicators						
Vitamin E insuficiency (<11 µmol/L)	3.03	2.00, 4.57	<0.001	2.63	1.76, 3.93	<0.001
Serum folate (median, nmol/L)						
<23.6	*Reference*			*Reference*		
≥23.6	0.66	0.45, 0.98	0.042	0.70	0.48, 1.03	0.076

PR, Prevalence Ratio; Models were adjusted for sex, age and race/ethnicity; * Adjusted for other variables in the same or higher levels following the hierarchical conceptual framework.
